# Neurochemical and Behavioral Effects of a New Hallucinogenic Compound 25B-NBOMe in Rats

**DOI:** 10.1007/s12640-020-00297-8

**Published:** 2020-12-18

**Authors:** Adam Wojtas, Monika Herian, Mateusz Skawski, Małgorzata Sobocińska, Alejandro González-Marín, Karolina Noworyta-Sokołowska, Krystyna Gołembiowska

**Affiliations:** Maj Institute of Pharmacology, Polish Academy of Sciences, Department of Pharmacology, 12 Smętna, 31-343 Kraków, Poland

**Keywords:** 25B-NBOMe, Hallucinogen, Neurotransmitters release, Behavior, Genotoxicity

## Abstract

4-Bromo-2,5-dimethoxy-N-(2-methoxybenzyl)phenethylamine (25B-NBOMe) is a hallucinogen exhibiting high binding affinity for 5-HT_2A/C_ serotonin receptors. In the present work, we investigated its effect on dopamine (DA), serotonin (5-HT), acetylcholine (ACh), and glutamate release in the rat frontal cortex, striatum, and nucleus accumbens. Hallucinogenic activity, impact on cognitive and motor functions, and anxiogenic/anxiolytic properties of this compound were also tested. The release of DA, 5-HT, ACh, and glutamate was studied using microdialysis in freely moving animals. Hallucinogenic activity was investigated using head and body twitch response (WDS), cognitive functions were examined with the novel object recognition test (NOR), locomotor activity was studied in the open field (OF), while anxiogenic/anxiolytic effect was tested using the light/dark box (LDB). Neurotoxicity was evaluated with the comet assay. 25B-NBOMe increased DA, 5-HT, and glutamate release in all studied brain regions, induced hallucinogenic activity, and lowered the recognition index (*Ri*) vs. control in the NOR test. It also decreased locomotor activity of rats in the OF test. The effect of 25B-NBOMe in the NOR test was inhibited by scopolamine. In the LDB test, the time spent in the dark zone was longer in comparison to control and was dose-dependent. In contrast to MDMA, 25B-NBOMe showed subtle genotoxic effect observed in the comet assay.

Our findings indicate that 25B-NBOMe shows hallucinogenic activity in the wide range of doses. The changes in neurotransmitter levels may be related to 25B-NBOMe affinity for 5-HT_2A_ receptor. Alterations in the NOR, OF, and LDB indicate that 25B-NBOMe impacts short-term memory, locomotion, and may be anxiogenic.

**Electronic Supplementary Material:**

The online version of this article (10.1007/s12640-020-00297-8) contains supplementary material, which is available to authorized users.

## Introduction

It is without a doubt that humans have been using hallucinogens for ages; along with ethanol, they are the oldest psychoactive substances known to mankind (Nichols 2016; Schultes et al. 2001). They are known to induce powerful visual and auditory hallucinations, alter perception, and have profound effect on the users’ mood (Nichols 2016).

Classical hallucinogens can be divided by their structure into 2 main categories: indoleamines, e.g., DMT (N,N-dimethyltryptamine) or LSD (lysergic acid diethylamide), and phenylalkylamines, e.g., mescaline or DOI (2,5-dimethoxy-4-iodoamphetamine) (Nichols 2012). While the former demonstrate affinity for nearly all subgroups of 5-HT receptors, the latter bind mainly to the 5-HT_2_ receptor family (Pierce and Peroutka 1989; Titeler et al. 1988). Holistic data gathered from many studies indicate that hallucinogens exert their psychoactive effects via activation of the cortical 5-HT_2A_ receptors (Glennon et al. 1984; Marek and Aghajanian 1996; Sipes and Geyer 1995; Wing et al. 1990). All hallucinogens produce head twitch response (HTR) in rodents, a phenomenon that is parallel to hallucinations in humans, as this effect can be a factor differentiating hallucinogenic from non-hallucinogenic 5-HT_2A_ receptor agonists (González-Maeso et al. 2007; Halberstadt and Geyer 2013). Studies conducted in 5-HT_2A_ receptor knockout mice have proven that the activation of this receptor is obligatory for the induction of head twitches (González-Maeso et al. 2007; Halberstadt and Geyer 2013).

Even though the “classical” serotoninergic hallucinogens (known also as psychedelics) rarely induce any form of toxicity (Nichols 2016), recently, a new group of synthetic hallucinogens emerged, which are responsible for a plethora of cases of heavy poisoning and fatalities (Baumann et al. 2017; Shanks et al. 2015; Walterscheid et al. 2014). These drugs are the N-(2-methoxybenzyl)-2,5-dimethoxy-4-substituted phenethylamines (NBOMe). The NBOMe compounds are phenethylamine derivatives which undergo the process of N-benzylation, which greatly increases both their affinity and efficacy at the 5-HT_2A_ receptor (Baumann et al. 2017). They have become available to drug users since they first appeared on the drug market around 2010.

One of the first representatives of this group 2-(4-bromo-2,5-dimethoxyphenyl)-N-[(2-methoxyphenyl)methyl]ethanamine (25B-NBOMe) was synthesized by Ralf Heim in 2003 (Heim 2003). It is characterized by a very high, subnanomolar affinity for the 5-HT_2A_ receptor (*Ki* = 0.5 nM) and activation potency (*EC*_*50*_ = 40 nM) (Rickli et al. 2015), which results in its strong psychoactive properties (Papoutsis et al. 2015). What is more, it seems to strongly affect dopaminergic system; it can induce conditioned place preference at certain doses in mice, and it induces self-administration in rats, which also suggests that it may exhibit reinforcing properties (Custodio et al. 2019; Miliano et al. 2019). What is more, it increases dopamine D_1_ receptor levels in the nucleus accumbens, while decreasing dopamine D_2_ receptor levels, and decreases dopamine transporter (DAT) levels in the ventral tegmental area (VTA) (Custodio et al. 2019).

Serotonin 5-HT_2A_ receptors are widely distributed in the CNS, especially in the rat medial prefrontal cortex (mPFC) where they regulate functions of pyramidal projection neurons (Nocjar et al. 2015). In addition, the presence of 5-HT_2A_ receptors was also evidenced in the basal ganglia, especially in the nucleus accumbens and caudate nucleus (Zhang and Stackman 2015). 5-HT_2A_ receptors in cortico-striatal projection are involved in impairment of attention (Carli and Invernizzi 2014) and may play a role in restoration of motor function resulting from dopamine (DA) depletion (Ansah et al. 2011). Interestingly, clinical studies suggest that naturally occurring classical hallucinogens, such as DMT, psilocybin, and pharmacologically related LSD may be used to treat drug dependence, anxiety, and mood disorders (dos Santos et al. 2016).

Up to date, limited data have been gathered regarding NBOMes and their pharmacology. In our recent work, we have demonstrated that another NBOMe compound, 25I-NBOMe, having similar in vitro affinity for 5-HT_2A_ receptor, affected DA, serotonin (5-HT), and glutamatergic neurotransmission and showed hallucinogenic activity (Herian et al. 2019).

The aim of this study was to assess in vivo 25B-NBOMe effect on brain neurotransmission and rat behavior. Microdialysis in the rat frontal cortex, striatum, and nucleus accumbens was performed to determine the influence of the 25B-NBOMe on DA, 5-HT, glutamate, and acetylcholine (ACh) levels. To assess the cognitive effects of 25B-NBOMe, the novel object recognition test (NOR) was performed. To estimate 25B-NBOMe hallucinogenic effect resulting from 5-HT_2A_ receptor stimulation, head and body twitch response was observed. The effect of 25B-NBOMe on anxiety was measured using the light/dark box test (LDB). Additionally, locomotor activity was measured in the open field test (OF). Putative genotoxic effect was also tested using the comet assay.

## Materials and Methods

### Animals

Male Wistar-Han rats (Charles River, Sulzfeld, Germany) weighting from 280 to 350 g (age of 120–180 PND) were used in all performed experiments. The animals were initially acclimatized and housed (5 per cage) in environmentally controlled rooms under 12-h light/dark cycle (the light was switched on at 6 a.m.) at a temperature of 23 ± 1 °C and humidity of 55 ± 10%. Rats had free access to typical laboratory food and tap water (VRF 1, Special Diets Services, Witham, UK), enriched environment was not applied. The studies strictly conformed to European regulations for animal experimentation (EU Directive 2010/63/EU on the protection of animals used for scientific purposes). The experimental protocols were approved by the Local Ethics Commission for Experimentation on Animals (permit number: 186/2017, 188/2017, 189/2017). This article does not contain any studies with human participants by any of the authors.

### Drugs and Reagents

2-(4-Bromo-2,5-dimethoxyphenyl)-N-(2-methoxybenzyl)-ethanamine (25B-NBOMe) was purchased from Cayman Chemical Company (Michigan, USA), while MDMA from Toronto Research Chemicals Inc. (Canada). LSD and scopolamine came from Sigma-Aldrich (Poland). All necessary chemicals for analysis with the use of high-performance liquid chromatography (HPLC) were obtained from Merck (Warszawa, Poland) and were of the highest purity. O-phthalaldehyde (OPA) obtained from Sigma-Aldrich was used for derivatization of glutamate to electroactive compound. The chemicals used for the comet assay were from Trevigen (Gaithersburg, MD, USA).

### Drug Administration

During the experiment animals received subcutaneous (*sc*) single injections of 25B-NBOMe dissolved in 0.9% NaCl at five doses of 0.1, 0.3, 1, 3, and 10 mg/kg. The subcutaneous injection of 25B-NBOMe was chosen because it seems to be a favorable way of administration in the case of this group of compounds, in comparison to intraperitoneal injection, as shown by Baumann et al. (2017). MDMA (10 mg/kg) was also dissolved in 0.9% NaCl and was injected intraperitoneally (*ip*). LSD was injected *ip* at a dose of 0.1 mg/kg. Scopolamine dissolved in 0.9% NaCl was injected *ip* at the dose of 1 mg/kg 5 min before administration of 25B-NBOMe. The control group was administered with 0.9% NaCl solution in the same way.

### Brain Microdialysis

Ketamine and xylazine solutions at doses of 75 mg/kg and 10 mg/kg, respectively, were used to anesthetize animals. Microdialysis probes (MAB 4.15.3Cu, MAB 4.15.4Cu, and MAB 4.15.2Cu AgnTho’s AB, Lindingö, Sweden) were implanted into the rat frontal cortex, striatum, and nucleus accumbens using the following coordinates (mm): AP + 2.7, L + 0.8, V − 6.5, from the dura (frontal cortex); AP + 1.2, L + 0.8, V − 7.0 (striatum); and AP + 1.6, L + 1.0, V − 8.0 (n. accumbens) (Paxinos and Watson 1998). Each group contained six animals. On the next day, probe inlets were connected to a syringe pump (BAS, West Lafayette, IN, USA), delivering artificial cerebrospinal fluid composed of (mM) 147 NaCl, 2.7 KCl, 1.0 MgCl_2_, 1.2 CaCl_2_; pH 7.4 at a flow rate of 2 μL/min. After 2 h of washout period, five basal dialysate samples were collected every 20 min, then animals were injected subcutaneously with 25B-NBOMe as indicated in the figure captions and fraction collection continued for 240 min. At the end of the experiment, the rats were sacrificed and their brains were histologically verified for the proper probe placement.

### Extracellular Concentration of DA, 5-HT, and Glutamate

The DA and 5-HT concentrations in dialysate fractions were analyzed by HPLC with electrochemical detection. Chromatography was performed using an Ultimate 3000 System (Dionex, Sunnyvale, CA, USA), electrochemical detector Coulochem III (model 5300; ESA, Chelmsford, MA, USA) with a 5020 guard cell, a 5040 amperometric cell, and a Hypersil Gold C18 analytical column (3 μm, 100 × 3 mm; Thermo Fischer Scientific, Waltham, MA, USA). The mobile phase was composed of 0.1 M potassium phosphate buffer adjusted to pH 3.8, 0.5 mM Na_2_EDTA, 100 mg/L 1-octanesulfonic acid sodium salt, and 2% methanol. The flow rate during analysis was set at 0.6 mL/min. The applied potential of a guard cell was 600 mV, while that of amperometric cell was 300 mV with a sensitivity set at 10 nA/V. The chromatographic data were processed by Chromeleon v.6.80 (Dionex) software package run on a personal computer. The limit of detection of DA and 5-HT in dialysates was 0.002 pg/10 μL for DA and 0.01 pg/10 μL for 5-HT.

Glutamate levels in the extracellular fluids were measured electrochemically after derivatization with OPA/sulfite reagent to form isoindole-sulfonate derivative (Rowley et al. 1995). Chromatography was performed using an Ultimate 3000 pump (Dionex), LC-4B amperometric detector with a cross-flow detector cell (BAS, IN, USA), and a HR-80 column (3 μm, 80 × 4.6 mm; ESA Inc, Chelmsford, MA, USA). The mobile phase consisted of 100 mM monosodium orthophosphate at pH 4.6 and 4% methanol. The flow rate was 1 mL/min, and the applied potential of a 3-mm glassy carbon electrode was set at + 600 mV at a sensitivity of 5 nA/V. Glutamate-derivative peak was compared with the respective standard, and the data were processed using Chromax 2005 (Pol-Lab, Warszawa, Poland) software on a personal computer. The limit of detection of glutamate in dialysates was 0.03 ng/10 μL.

### Extracellular Concentration of ACh

Extracellular levels of ACh were analyzed by UHPLC with electrochemical detection. The ACh analysis is based on ion-pairing HPLC separation, followed by online enzymatic conversion of ACh to hydrogen peroxide, and detection on a Pt working electrode (SenCell with 2 mm Pt working electrode) and HyREF reference electrode at the potential of 200 mV. Chromatography was performed using the ALEXYS Neurotransmitter Analyzer, a DECADE Elite electrochemical detector, AS 110 Autosampler, and LC 110 pump (Antec Leyden B. V., Zoeterwoude, The Netherlands). ACh as positively charged was separated on Acquity UPLC HSS T3 analytical column (1.8 μm, 1 × 50 mm; Waters, Milford, MA, USA). After separation, ACh passed through an immobilized enzyme reactor AChE/ChOx IMER (AC-ENZYM II, 1 × 4 mm, Eicom, Kyoto, Japan). The mobile phase was composed of 50 mM monosodium orthophosphate buffer adjusted to pH 7.8, 0.5 mM Na_2_EDTA, 2.8 g/L 1-octanesulfonic acid sodium salt, and 0.5 mM tetramethylammonium chloride. The flow rate during analysis was set to 0.05 mL/min. The chromatographic data were processed by CLARITY v.6.2.0.208 (DataApex Ltd.) chromatography software run on a personal computer. The limit of detection of ACh in dialysates was 0.0037 pg/10 μL.

### Head and Body Shakes Test

The behavior defined as rapid shaking of the head, neck, and trunk from one side to the other, analogous to a wet dog shaking (WDS) to dry itself (Klein et al. 2018) was counted immediately after drug injection during 240-min observation period by an experienced observer who was blind to the treatments. Scores were summed and totaled from 12 observation periods. Results were expressed as an average of sum values of all episodes during the observation time.

### Novel Object Recognition Test

The procedure of the NOR test was adopted from Antunes and Biala (2012) and Orzelska-Gorka et al. (2016). Apparatus consisted of a wooden closed square arena with painted black walls (60 × 60 × 40 cm) illuminated with a bright white light (150 lx) focused on the center. Each animal was familiarized with the arena (pre-test, without any object) 24 h before the testing day. The animals were habituated to dimly lit experimental room at least 1 h before the procedure. The NOR test consisted of two sessions: introductory and recognition one (5 min each) with a 30 min inter-session interval. The introductory session was performed 20 min after injection of a drug with two identical objects (A1 and A2) situated in opposite corners, approximately 15 cm from the walls of the arena. In the recognition session, one object was replaced with a novel one (A = familiar, B = novel). The objects were a black metal can and a green vase, which were used interchangeably as a novel object in each experimental group. Location of a novel object in the recognition session was randomly assigned to each rat. The arena and the objects were cleaned after each session. Exploration of an object was defined as follows: licking, sniffing, or touching the object but not as sniffing, standing, sitting on the object or leaning against it. Exploration time was measured using a digital laboratory timer by two independent observers blind to the experimental design. Recognition index (*Ri*) was calculated using the equation:

*Ri* = $$\frac{\mathrm{Time\;spent\;on\;novel\;object\;exploration }\times 100}{\mathrm{Time\;spent\;on\;novel\;object\;exploration }+\mathrm{ time\;spent\;on\;familiar\;object\;exploration}}$$,

for results obtained during recognition session. It is considered to be an index of exploration of a novel object relative to the total exploration of both objects. The “*Ri*” ratio over 50% was defined as a successful recognition.

### Open Field Test

The open field test was performed as a modification of the procedure described by Rogóż and Skuza (2011). A round black arena (1 m in diameter) was used, which was virtually divided into 8 sections of the wheel. The test was conducted in the dimly lit room, except the middle of the arena, which was illuminated by 75-W light bulb placed 75 cm above it. Rats were placed in the middle of the arena 30 min after 25B-NBOMe subcutaneous injection, and their behavior was recorded for 10 min. The exploration was quantified with the following parameters: time of walking, number of line crossings, episodes of peeping under the arena, number of events of grooming, and number of rearings.

### Light/Dark Box Test

The light/dark box (LDB) test was performed in 4 computer-controlled Seamless Open Field Arenas for rats (43 × 43 × 30 cm; Med Associates; St Albans, Vermont, USA) that have 16 infrared emitters and photodetectors on each side of the box. The procedure from Noworyta-Sokolowska et al. (2019) was adapted to the present experimental design. A dark insert was used to divide the chamber into two equally sized compartments: a light compartment and a dark compartment. A hole in the insert enabled rats to move freely between compartments. The rat was placed in the dark compartment 20 min after injection of a drug and was allowed to explore freely for 15 min. The measured parameters included immobility time, ambulatory distance, vertical activity time, stereotypical activity time, and the time spent in the dark and light compartment. The data were collected using Med State software (Activity monitor, Med Associates).

### Alkaline Comet Assay

The alkaline comet assay was performed with the use of CometAssay® Reagent Kit for Single Cell Gel Electrophoresis Assay (Trevigen, Inc., Gaithersburg, MD, USA). At 72 h after injection of 25B-NBOMe, animals were sacrificed by decapitation and the frontal cortex was dissected. Fresh tissue placed on ice was used to isolate a nuclear fraction. After homogenization and several purification and centrifugation stages (as described previously in Noworyta-Sokołowska et al. 2019), nuclear suspension was obtained using a sucrose gradient (2.8 M/2.6 M, bottom to top). The nuclear fraction was mixed with low melting point agarose and transferred immediately onto CometSlides™. The following steps were carried out in accordance with Trevigen CometAssay® protocol: membrane lysis, DNA unwinding, alkaline electrophoresis, and staining (SYBR® Gold). Stained slides were examined under a fluorescence microscope (NIKON Eclipse 80i, NIKON Instruments Inc., Melville, NY, USA). The data was analyzed using OpenComet software v.1.3, a plugin of ImageJ program v.1.47 (NIH, Bethesda, MD, USA). DNA damage was presented as a tail moment. Tail moment incorporates a measure of both the smallest detectable size of migrating DNA (reflected by the comet tail length) and the number of damaged pieces (represented by the intensity of DNA in the tail).

### Data Analysis

Drug effects on DA, 5-HT, ACh, and glutamate release in the brain regions were analyzed with repeated measures ANOVA followed by Tukey’s post hoc test. All obtained data were presented as a percent of the basal level assumed to be 100%. The data from the wet dog shake test, the novel object recognition test, and locomotor behavior of rats in the open field were analyzed using one-way ANOVA followed by Tukey’s post hoc test and the *t* test where appropriate. Data collected from the alkaline comet assay were analyzed with the *t* test, while the light/dark box test was analyzed using Mann–Whitney’s test. The differences were considered significant if *p* value was smaller than 0.05. All statistical analyses were carried out using STATISTICA v.10 StatSoft Inc. 1984–2011 (San Francisco, CA, USA) and GraphPad Prism v.5.00 GraphPad Software Inc. (La Jolla, CA, USA).

## Results

### The Effect of 25B-NBOMe Administration on the Extracellular Levels of DA, 5-HT, ACh, and Glutamate in the Rat Frontal Cortex

**Fig. 1:**
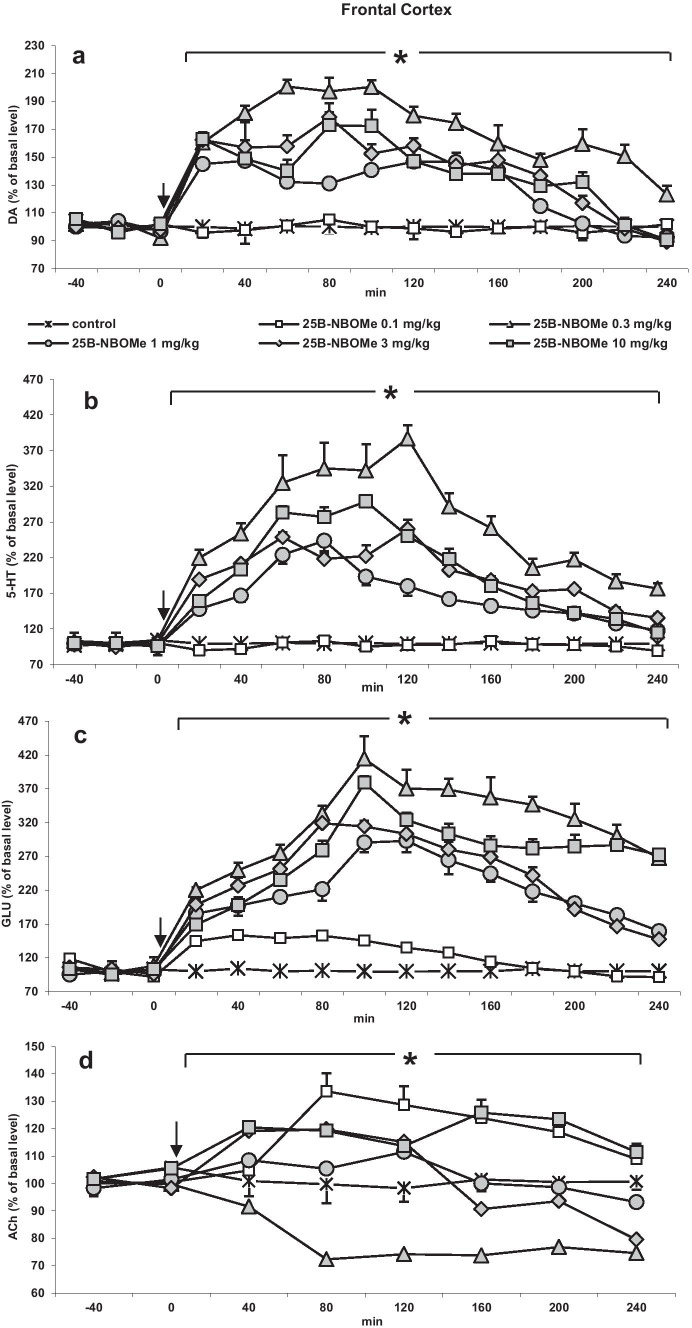
The time-course effect of 25B-NBOMe on extracellular levels of **a** dopamine (DA), **b** serotonin (5-HT), **c** glutamate (GLU), and **d** acetylcholine (ACh) in the rat frontal cortex. Values are the mean ± standard error of the mean (SEM), *n* = 6 per experimental group. For ACh measurements, two consecutive dialysate fractions were pooled. The time of drug injection is indicated by an arrow. The basal extracellular levels were as follows: for DA, 0.99 ± 0.06 nM, *n* = 30; for 5-HT, 0.19 ± 0.008 nM, *n *= 30; for ACh, 36.5 ± 3.93 nM, *n* = 30; for GLU, 1.76 ± 0.16 μM, *n* = 30; *p < 0.0002 vs. control group (repeated measures ANOVA and Tukey’s post hoc test)

25B-NBOMe significantly (*p* < 0.0002) increased DA levels in the rat frontal cortex. The dose of 0.3 mg/kg was the most potent while the doses of 1, 3, and 10 mg/kg were less potent, but their effect was still significant in comparison to control group. The dose of 0.1 mg/kg did not increase cortical extracellular DA level (Fig. 1a). Repeated measures ANOVA showed a significant effect of treatment groups (*F*_5,30_ = 266, *p* < 0.0001), sampling period (*F*_11,330_ = 130, *p* < 0.0001), and the interaction between treatment groups and sampling period (*F*_55,330_ = 20, *p* < 0.0001). Tukey’s post hoc tests showed significant difference in dialysate DA levels (*p* < 0.001) between doses of 0.1, 1, 3, and 10 mg/kg compared with the effect of 0.3 mg/kg 25B-NBOMe dose.

The extracellular 5-HT level was increased the most by the dose of 0.3 mg/kg, and the doses of 1, 3, and 10 mg/kg were less active but still significant (*p* < 0.0002). 25B-NBOMe at the dose of 0.1 mg/kg did not change extracellular 5-HT level (Fig. 1b). Repeated measures ANOVA showed a significant effect of treatment groups (*F*_5,30_ = 134, *p* < 0.0001), sampling period (*F*_11,330_ = 68, *p* < 0.0001), and the interaction between treatment groups and sampling period (*F*_55,330_ = 9.7, *p* < 0.0001). Tukey’s post hoc tests showed significant difference in dialysate 5-HT levels (*p* < 0.001) between doses of 0.1, 1, 3, and 10 mg/kg with respect to the effect of 0.3 mg/kg 25B-NBOMe dose.

The 25B-NBOMe dose of 0.3 mg/kg was also the strongest in increasing extracellular glutamate level. The effect of the remaining doses was less potent, yet still significant (*p* < 0.0002) (Fig. 1c). Repeated measures ANOVA showed a significant effect of treatment groups (*F*_5,30_ = 241, *p* < 0.0001), sampling period (*F*_11,330_ = 73, *p* < 0.0001), and the interaction between treatment groups and sampling period (*F*_55,330_ = 12.6, *p* < 0.0001). Tukey’s post hoc test showed significant difference in dialysate glutamate levels (*p* < 0.001) of doses 0.1, 1, 3, and 10 mg/kg with respect to effect of 0.3 mg/kg 25B-NBOMe dose.

The 25B-NBOMe doses of 0.1 and 10 mg/kg increased extracellular ACh level with similar potency (*p* < 0.001), the dose of 0.3 mg/kg significantly decreased ACh level (*p* < 0.001), while doses of 1 and 3 mg/kg were not effective (Fig. 1d). Repeated measures ANOVA showed a significant effect of treatment groups (*F*_5,30_ = 137, *p* < 0.0001), sampling period (*F*_5,150_ = 30, *p* < 0.0001), and the interaction between treatment groups and sampling period (*F*_25,150_ = 16, *p* < 0.0001). Tukey’s post hoc tests showed a significant difference in dialysate ACh levels (*p* < 0.001) of doses of 0.1, 1, 3, and 10 mg/kg with respect to the effect of 0.3 mg/kg 25B-NBOMe dose.

The total effect of 25B-NBOMe on extracellular levels of DA, 5-HT, glutamate, and ACh in the rat frontal cortex calculated as an area under the curve (AUC) and expressed as the percent of each basal level is presented in Fig. 4a.

### The Effect of 25B-NBOMe Administration on the Extracellular Levels of DA, 5-HT, ACh, and Glutamate in the Rat Striatum and Nucleus Accumbens

**Fig. 2:**
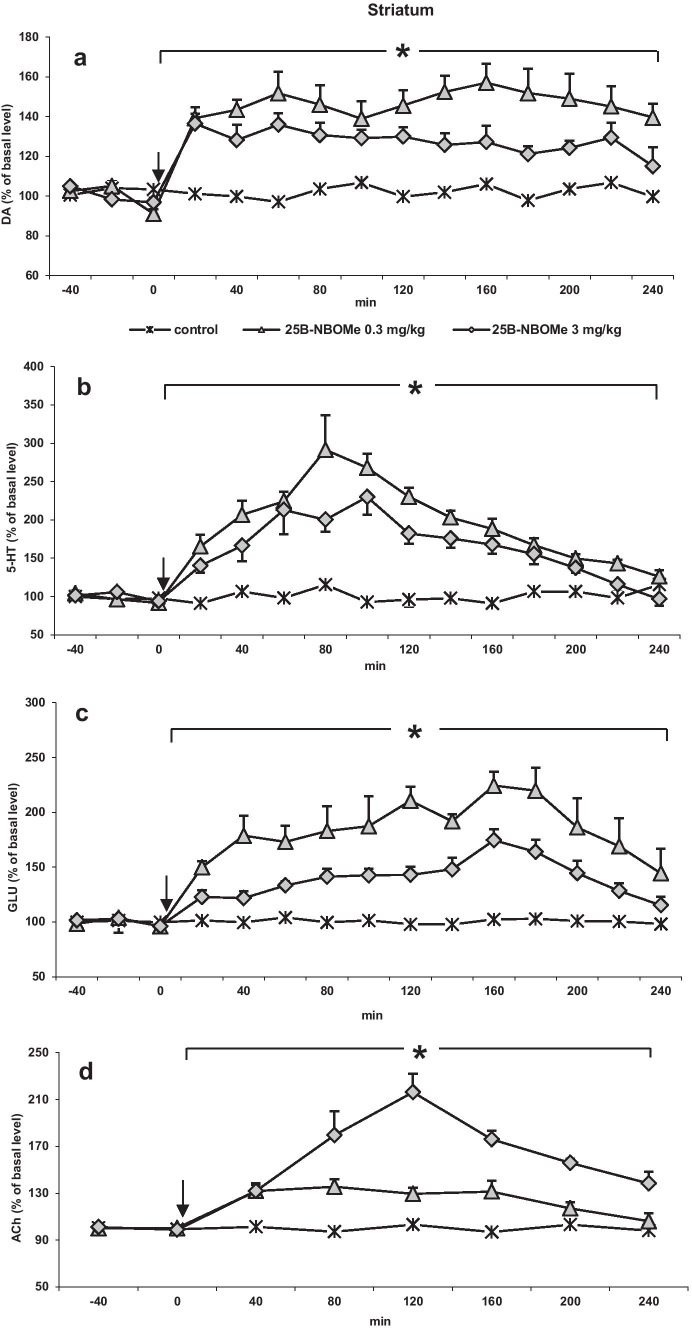
The time-course effect of 25B-NBOMe on extracellular levels of **a** dopamine (DA), **b** serotonin (5-HT), **c** glutamate (GLU), and **d** acetylcholine (ACh) in the rat striatum. Values are the mean ± standard error of the mean (SEM), *n* = 6 per experimental group. For ACh measurements, two consecutive dialysate fractions were pooled. The time of drug injection is indicated by an arrow. The basal extracellular levels were as follows: for DA, 5.17 ± 0.28 nM, *n* = 30; for 5-HT, 0.27 ± 0.014 nM, *n* = 30; for ACh, 48.9 ± 3.64 nM, *n* = 30; for GLU, 2.84 ± 0.22 μM, *n* = 30; *p < 0.0002 vs. control group (repeated measures ANOVA and Tukey’s post hoc test)

The doses of 0.3 mg/kg (more selective) and 3 mg/kg (less selective in activation of 5-HT_2A_ receptors) of 25B-NBOMe were chosen for microdialysis experiments in the striatum and nucleus accumbens to minimize the number of animals. Both 25B-NBOMe doses increased extracellular levels of DA, 5-HT, glutamate, and ACh in the rat striatum (Fig. 2 a, b, c, and d). The dose of 0.3 mg/kg produced a larger increase in DA, 5-HT, and glutamate levels than the dose of 3 mg/kg, respectively. Repeated measures ANOVA showed an effect of treatment on DA levels (*F*_2,15_ = 94, *p* < 0.0001, time *F*_11,165_ = 3.69, *p* < 0.0001, time × treatment interaction *F*_22, 165_ = 3.74, *p* < 0.0001). Tukey’s post hoc tests showed a larger increase in dialysate DA in the striatum after 25B-NBOMe 0.3 and 3 mg/kg with respect to control values (*p* < 0.0002). Repeated measures ANOVA showed an effect of treatment on 5-HT levels (*F*_2,15_ = 333, *p* < 0.0001, time *F*_11,165_ = 8.5, *p* < 0.0001, time × treatment interaction *F*_22, 165_ = 3.03, *p* < 0.0003). Tukey’s post hoc tests showed a larger increase in dialysate 5-HT in the striatum after 25B-NBOMe 0.3 and 3 mg/kg with respect to control values (*p* < 0.0002). Repeated measures ANOVA showed an effect of treatment on glutamate levels (*F*_2,15_ = 339, *p* < 0.0001, time *F*_11,165_ = 21.65, *p* < 0.0001, time × treatment interaction *F*_22, 165_ = 6.32, *p* < 0.0001). Tukey’s post hoc tests showed a larger increase in dialysate glutamate in the striatum after 25B-NBOMe 0.3 and 3 mg/kg with respect to control values (*p* < 0.0002). The dose of 3 mg/kg most potently affected the ACh levels. Repeated measures ANOVA showed an effect of treatment on ACh (*F*_2,15_ = 58, *p* < 0.0001, time *F*_5,75_ = 12.30, *p* < 0.0001, time × treatment interaction *F*_10, 75_ = 7.80, *p* < 0.0001). Tukey’s post hoc tests showed a larger increase in dialysate ACh in the striatum with respect to control values (*p* < 0.0002).

**Fig. 3:**
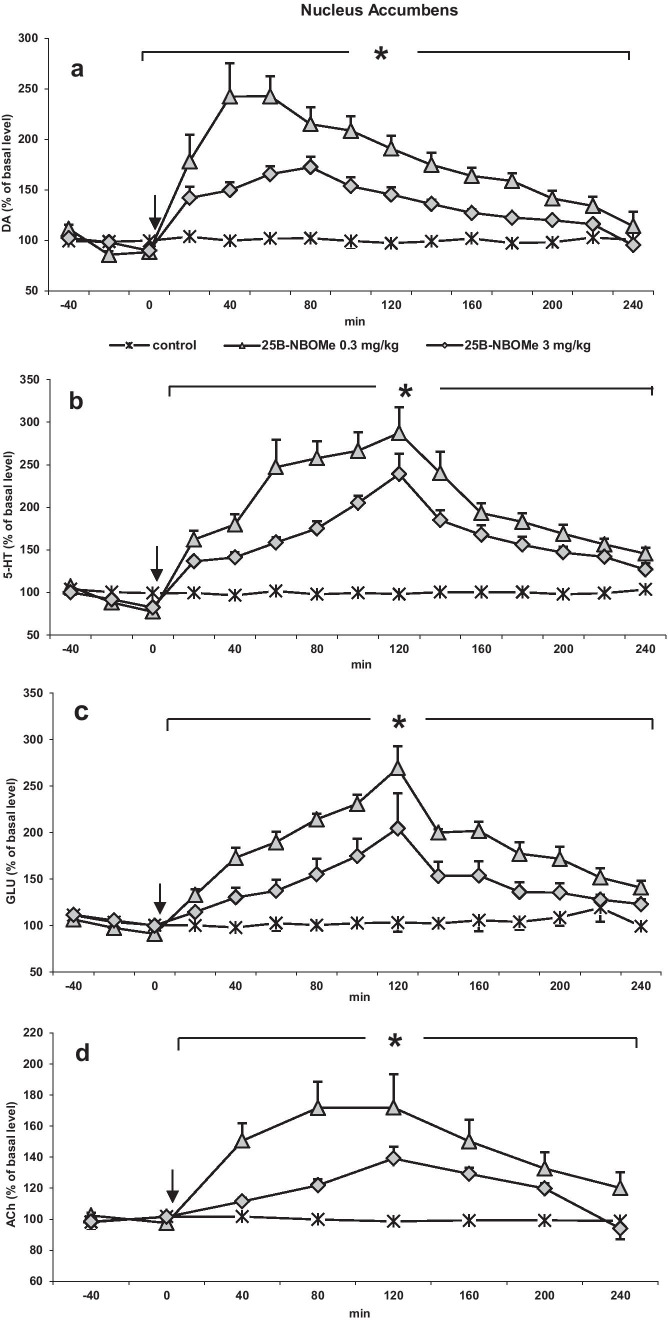
The time-course effect of 25B-NBOMe on extracellular levels of **a** dopamine (DA), **b** serotonin (5-HT), **c** glutamate (GLU), and **d** acetylcholine (ACh) in the rat nucleus accumbens. Values are the mean ± standard error of the mean (SEM), *n* = 6 per experimental group. For ACh measurements, two consecutive dialysate fractions were pooled. The time of drug injection is indicated with an arrow. The basal extracellular levels were as follows: for DA, 1.05 ± 0.059 nM, *n* = 30; for 5-HT, 0.199 ± 0.01 nM, *n* = 30; for ACh, 19.5 ± 1.34 nM, *n* = 30; for GLU, 2.10 ± 0.13 μM, *n* = 30; *p < 0.0002 vs. control group (repeated measures ANOVA and Tukey’s post hoc test)

Similarly to the striatum, 25B-NBOMe at doses of 0.3 and 3 mg/kg significantly (*p* < 0.0002) increased extracellular levels of DA, 5-HT, glutamate, and ACh in the rat nucleus accumbens (Fig. 3 a, b, c, and d), but the effect was not dose-dependent since the lower dose of 25B-NBOMe was more effective in increasing the release of all neurotransmitters. Repeated measures ANOVA showed an effect of treatment on DA levels (*F*_2,15_ = 337, *p* < 0.0001, time *F*_11,165_ = 49, *p* < 0.0001, time × treatment interaction *F*_22, 165_ = 18, *p* < 0.0001). Tukey’s post hoc tests showed a larger increase in dialysate DA in the nucleus accumbens after 25B-NBOMe 0.3 and 3 mg/kg with respect to control values (*p* < 0.0002). Repeated measures ANOVA showed an effect of treatment on 5-HT levels (*F*_2,15_ = 516, *p* < 0.0001, time *F*_11,165_ = 80, *p* < 0.0001, time × treatment interaction *F*_22, 165_ = 28, *p* < 0.0001). Tukey’s post hoc tests showed a larger increase in dialysate 5-HT in the nucleus accumbens after 25B-NBOMe 0.3 and 3 mg/kg with respect to control values (*p* < 0.0002). Repeated measures ANOVA showed an effect of treatment on glutamate levels (*F*_2,15_ = 81, *p* < 0.0001, time *F*_11,165_ = 67, *p* < 0.0001, time × treatment interaction *F*_22, 165_ = 21, *p* < 0.0001). Tukey’s post hoc tests showed a larger increase in dialysate glutamate in the nucleus accumbens after 25B-NBOMe 0.3 and 3 mg/kg with respect to control values (*p* < 0.0002). The extracellular ACh level was increased more strongly by the lower dose of 25B-NBOMe. Repeated measures ANOVA showed an effect of treatment on ACh levels (*F*_2,15_ = 201, *p* < 0.0001, time *F*_5,75_ = 31, *p* < 0.0001, time × treatment interaction *F*_10,75_ = 11, *p* < 0.0001). Tukey’s post hoc tests showed a larger increase in dialysate ACh in the nucleus accumbens after 25B-NBOMe 0.3 and 3 mg/kg with respect to control values (*p* < 0.0002).

**Fig. 4:**
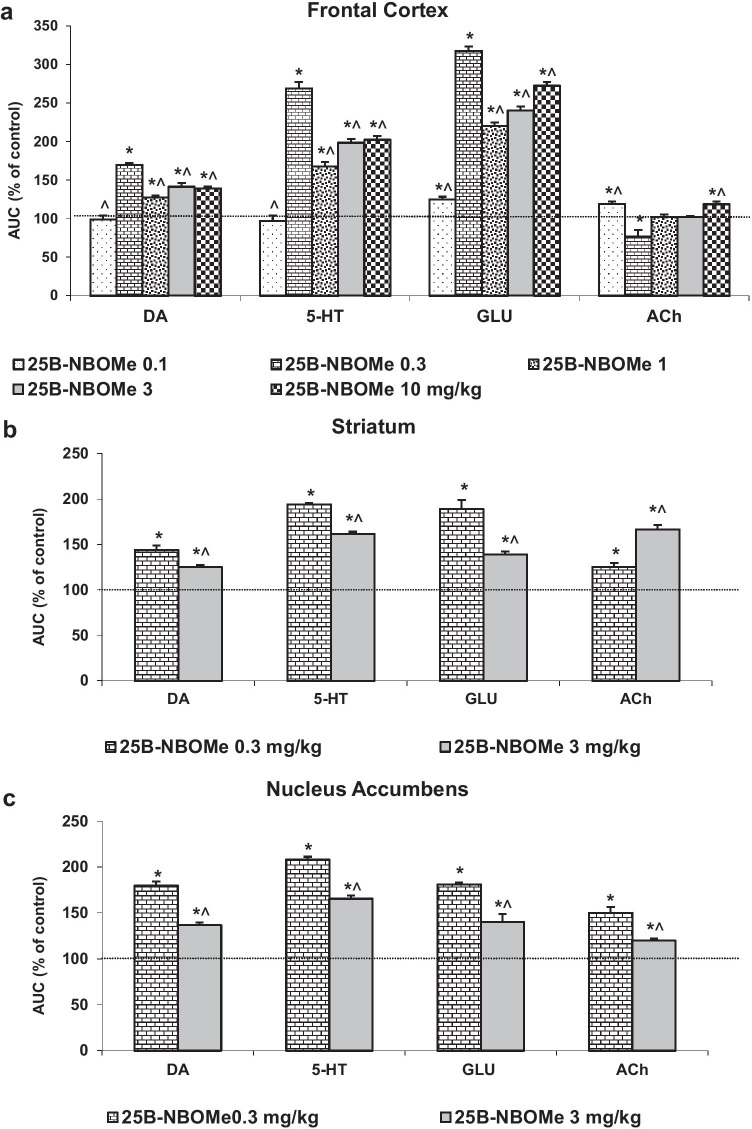
The total effect of 25B-NBOMe on extracellular levels of dopamine (DA), serotonin (5-HT), glutamate (GLU) and acetylcholine (ACh) in the rat frontal cortex (**a**), striatum (**b**), and nucleus accumbens (**c**) calculated as an area under the curve (AUC) and expressed as the percent of each basal level. Values are the mean ± standard error of the mean (SEM), *n* = 6 per experimental group. *p < 0.0002 vs. control group, ^*p* < 0.05 vs. 0.3 mg/kg (one-way ANOVA and Tukey’s post hoc test)

The total effect of 25B-NBOMe on extracellular levels of DA, 5-HT, glutamate, and ACh in the rat striatum and nucleus accumbens calculated as an area under the curve (AUC) and expressed as the percent of each basal level is presented in Fig. 4 b and c.

### The Effect of 25B-NBOMe in the Wet Dog Shake Test

**Fig. 5:**
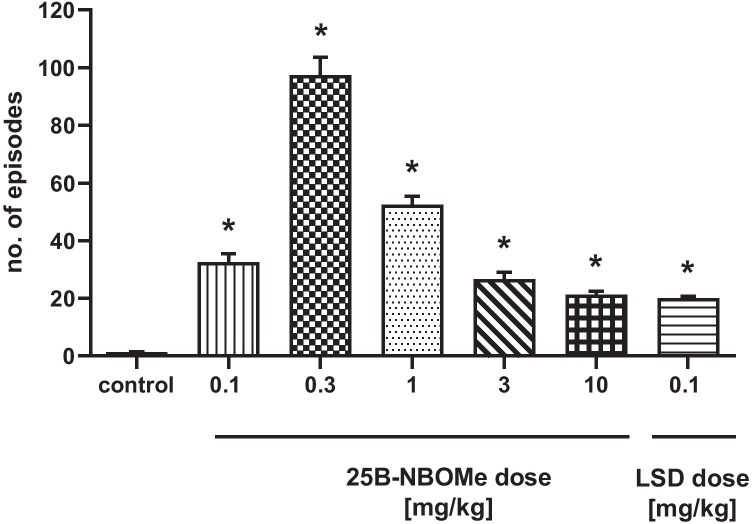
The effect of 25B-NBOMe and LSD on head and body twitches (WDS) in rats. The number of episodes counted for 240 min starting immediately after the injection is shown. Values are the mean ± standard error of the mean (SEM), *n* = 8 per experimental group. *p < 0.0001 vs. control group (one-way ANOVA and Tukey’s post hoc test)

25B-NBOMe induced head and body twitches (WDS) in rats, which were observed immediately after the administration. The dose of 0.3 mg/kg produced the most potent effect (Fig. 5). The effect of 0.1, 1, 3, and 10 mg/kg doses was weaker but significant (*F*_6,49_ = 127, *p* < 0.0001). LSD at a dose of 0.1 mg/kg was nearly equally potent as the highest dose of 25B-NBOMe in inducing the hallucinogenic response.

### The Effect of 25B-NBOMe on Performance of Rats in the Novel Object Recognition Test

In this and the other behavioral experiments, we evaluated the effect of the two doses of 25B-NBOMe (0.3 and 3 mg/kg, sc). The results of this study showed an inverted “U”-shaped dose–response effect of 25B-NBOMe in increasing extracellular DA, 5-HT, and glutamate levels and in hallucinogenic activity in rats. The dose of 0.3 mg/kg was the most potent, while responses of higher doses were weaker. Therefore, for other behavioral tests, these two doses were chosen; the dose of 0.3 mg/kg representing more selective response resulting from activation of 5-HT_2A_ receptors and less selective response to the dose of 3 mg/kg, possibly linked with activation of 5-HT_2C_ or 5-HT_1A_ receptors apart from 5-HT_2A_ receptors.

**Fig. 6:**
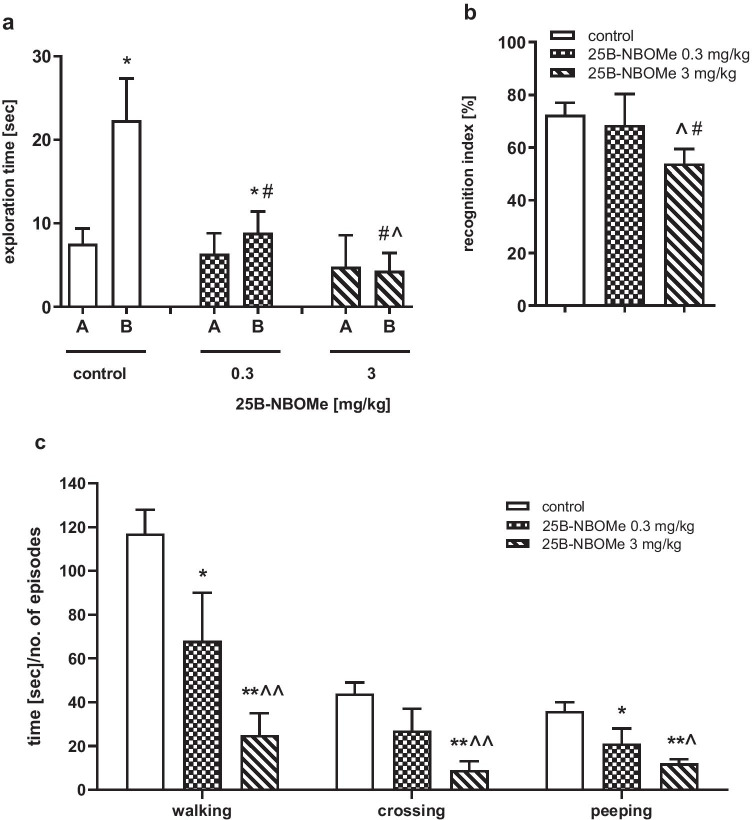
The effect of 25B-NBOMe (0.3 and 3 mg/kg) on performance of rats in the novel object recognition (**a**, **b**) test and on locomotor behavior of rats in the open field (**c**) test. **a** Exploration time in the recognition session for the familiar (A) and novel object (B). **b** *Ri* expressed as the time spent on novel object exploration in relation to the total exploration time of both novel and familiar objects. **c** The time spent on walking, the number of episodes of crossing and the number of episodes of peeping. Values are the mean ± standard error of the mean (SEM), *n* = 6–10 per experimental group. **a** *p < 0.05, **p < 0.01 novel vs. familiar object (*t* test); ^#^*p* < 0.01 vs. control (one-way ANOVA and Tukey’s post hoc test); **b** ^#^*p* < 0.01 vs. control; ^*p* < 0.01 vs. 25B-NBOMe 0.3 mg/kg (one-way ANOVA and Tukey’s post hoc test); **c** *p < 0.05, **p < 0.01 vs. control; ^*p* < 0.05; ^^*p* < 0.01 vs. 25B-NBOMe 0.3 mg/kg (one-way ANOVA and Tukey’s post hoc test)

The time of novel object exploration compared with familiar object exploration during the recognition session was observed to be increased in control group, and in the group treated with the lower but not the higher 25B-NBOMe dose (Fig. 6a). Statistical analysis of exploration time of the novel and familiar object during the recognition session showed a significant difference between control and both doses of 25B-NBOMe (Fig. 6a; *F*_2,23_ = 278, *p* < 0.0001). There was a significant difference in the *Ri* between animals treated with the higher dose of 25B-NBOMe and the control group (Fig. 6b; *F*_2,23_ = 139, *p* < 0.0001). Importantly, the *Ri* in the control and low-dose 25B-NBOMe-treated group reached ca. 72% and 68%, respectively, while in the 25B-NBOMe 3 mg/kg group, it was 45% (Fig. 6b).

**Fig. 7:**
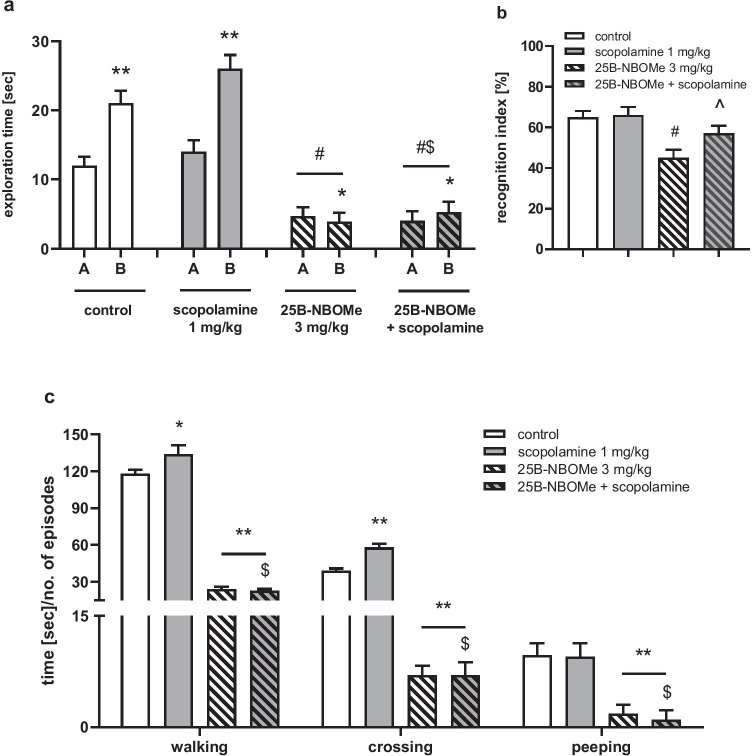
The effect of scopolamine (1 mg/kg) on 25B-NBOMe (3 mg/kg)-induced changes in rats’ performance in the novel object recognition (**a**, **b**) test and locomotor activity in the open field (**c**) test. **a** Exploration time in the recognition session for the familiar (A) and novel object (B). **b** *Ri* expressed as the time spent on novel object exploration in relation to the total exploration time of both the novel and familiar objects. **c** The time spent on walking, the number of episodes of crossing, and the number of episodes of peeping. Values are the mean ± standard error of the mean (SEM), *n* = 6–12 per experimental group. **a** *p < 0.05, **p < 0.01 novel vs. familiar object (*t* test); ^#^*p* < 0.01 vs. control; ^$^*p* < 0.01 vs. scopolamine (one-way ANOVA and Tukey’s post hoc test); **b** ^#^*p* < 0.01 vs. control; ^*p* < 0.01 vs. 25B-NBOMe 0.3 mg/kg (one-way ANOVA and Tukey’s post hoc test); **c** *p < 0.05, **p < 0.01 vs. control; ^$^*p* < 0.01 vs. scopolamine (one-way ANOVA and Tukey’s post hoc test)

Scopolamine (1 mg/kg, ip) increased the time of novel object exploration during the recognition session in comparison to control and to animals treated with 25B-NBOMe (3 mg/kg) (Fig. 7a). Statistical analysis of novel object exploration time in recognition session showed a significant difference between treatment groups (*F*_3,27_ = 40, *p* < 0.0001). There was also a reversal of 25B-NBOMe-induced decrease in *Ri* by scopolamine (Fig. 7b; *F*_3,27_ = 18, *p* < 0.01).

### The Effect of 25B-NBOMe on Locomotor Activity of Rats in the Open Field Test

25B-NBOMe decreased the time of walking (*F*_2,19_ = 134, *p* < 0.0001) and the number of episodes of crossing (*F*_2,19_ = 110, *p* < 0.0001) and peeping (*F*_2,19_ = 32, *p* < 0.0001) in a dose-dependent manner (Fig. 6c). No episodes of rearing and grooming were observed in animals treated with both doses of 25B-NBOMe (data not shown).

Scopolamine (1 mg/kg) increased the time of walking and the number of episodes of crossing in comparison to control, but had no effect on episodes of peeping (Fig. 7c) and did not affect the decrease in these parameters induced by 25B-NBOMe (3 mg/kg). Statistical analysis showed a significant effect of treatment groups on walking (*F*_3,23_ = 213, *p* < 0.0001), crossing (*F*_3,23_ = 164, *p* < 0.0001) and peeping (*F*_3,23_ = 68, *p* < 0.0001). No rearing and grooming behavior was observed (data not shown).

### The Effect of 25B-NBOMe on Anxiolytic/Anxiogenic-Like Activity in Rats in the Light/Dark Box Test

**Fig. 8:**
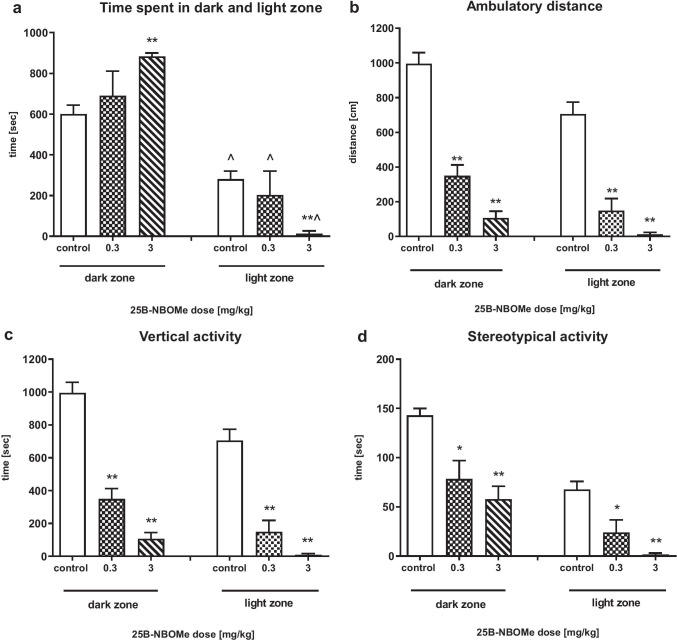
The effect of 25B-NBOMe (0.3 and 3 mg/kg) on activity of rats in the light/dark box test. **a** The time spent in the dark and light zone; **b**–**d** ambulatory distance, vertical, and stereotypical activity, respectively in the dark and light zone. Values are the mean ± standard error of the mean (SEM), *n* = 8 per experimental group. *p < 0.05, **p < 0.01 vs. control, ^*p* < 0.001 light vs. dark zone (Mann–Whitney’s test)

The time spent in the dark compartment was longer than in the light zone for all groups of animals (control U = 11, *p* < 0.001; 0.3 mg/kg U = 6, *p* < 0.001; 3 mg/kg U = 0, *p* < 0.001) (Fig. 8a); however, the time was longer and statistically significant in the rats treated with 25B-NBOMe at the dose of 3 mg/kg as compared to control (U = 0, *p* < 0.001). Accordingly, the time spent by all animals in the light zone was shorter and was decreased in the 25B-NBOMe 3 mg/kg group in comparison to control (U = 0, *p* < 0.001). 25B-NBOMe at doses of 0.3 and 3 mg/kg significantly decreased exploration of the dark and light zones. Exploration of the dark zone expressed as ambulatory distance (dark zone: U = 0, *p* < 0.001, U = 0, *p* < 0.001, light zone: U = 2, p < 0.001, U = 0, *p* < 0.001, respectively), vertical (dark zone: U = 0, *p* < 0.001, U = 0, *p* < 0.001, light zone: U = 2, *p* < 0.001, U = 0, *p* < 0.001, respectively) and stereotypical activity (dark zone: U = 15, *p* < 0.001, U = 0, *p* < 0.001, light zone: U = 15, *p* < 0.001, U = 0, *p* < 0.001, respectively) were significantly decreased by both doses of 25B-NBOMe in comparison to control (Fig. 8b, c, d).

### The Effect of 25B-NBOMe on Oxidative DNA Damage in the Rat Frontal Cortex

**Fig. 9:**
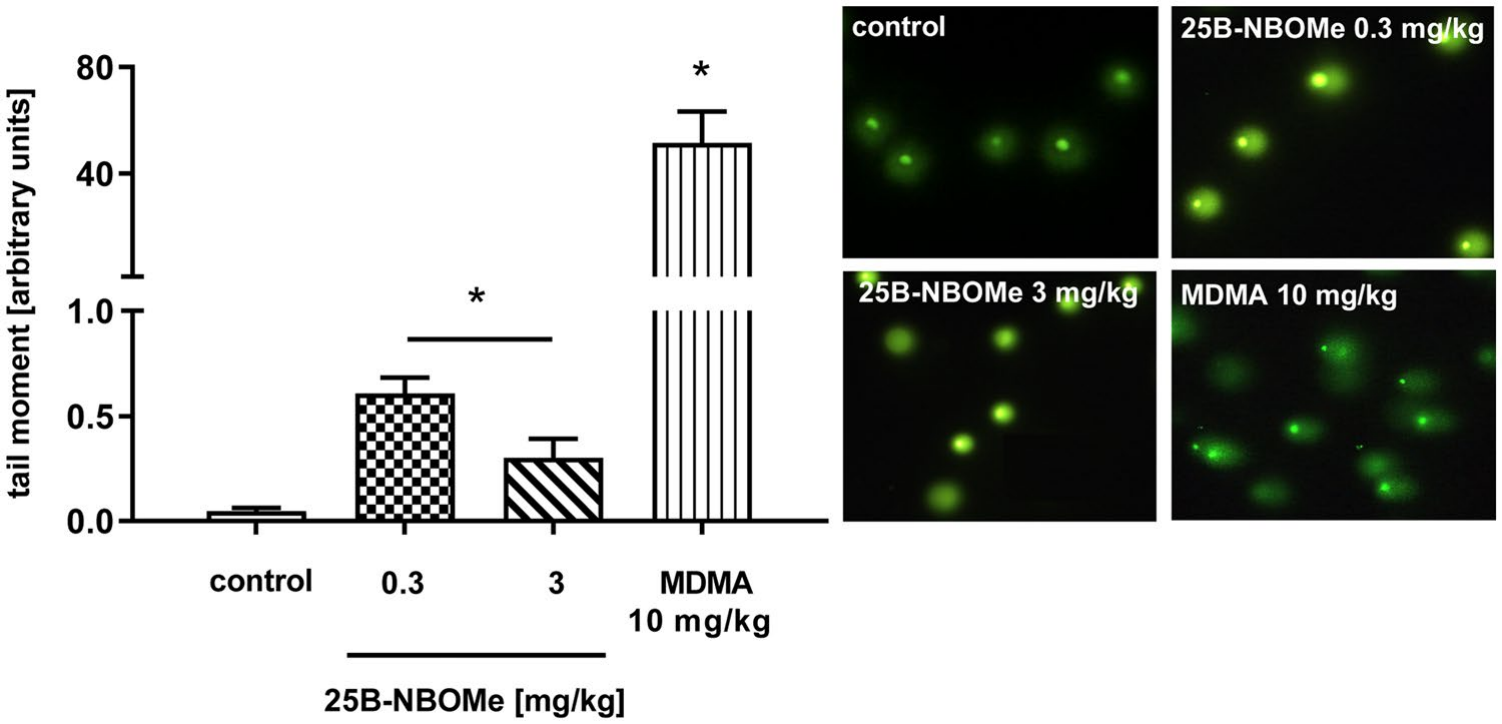
The effect of 25B-NBOMe (0.3, 3 mg/kg) and MDMA (10 mg/kg) on the oxidative damage of DNA in the nuclei from the rat frontal cortex. Data are the mean ± SEM (*n* = 6 animals per group) and represent tail moment shown as the product of the tail length and the fraction of total DNA in the tail. Typical microscopic images of nuclei from control, 25B-NBOMe- and MDMA-treated rats; *p < 0.01 in comparison to control group (*t* test). DNA damage is presented in arbitrary units

In contrast to MDMA (10 mg/kg), 25B-NBOMe given at doses of 0.3 and 3 mg/kg produced a minor DNA damage in the rat frontal cortex, shown as a tail moment as measured at 72 h after administration (Fig. 9). The low dose of 25B-NBOMe of 0.3 mg/kg markedly and a dose of 3 mg/kg less potently but still significantly damaged DNA in the rat frontal cortex. The effect of 25B-NBOMe was inversely correlated with the dose, but it was much weaker than that produced by MDMA. We also observed that 72 h after the treatment with 25B-NBOMe in the dose of 0.3 mg/kg a vast amount of nuclear DNA was damaged (95%), like after the treatment with 10 mg/kg of MDMA (99%). However, the level of the 25B-NBOMe-induced damages was by ca. 85 times lower than after MDMA as presented in Fig. 9. Instead, the dose of 3 mg/kg induced small and less frequent (18%) damages in comparison to the 25B-NBOMe in a dose of 0.3 mg/kg.

## Discussion

### Hallucinogenic Activity

The present study demonstrates that 25B-NBOMe is an extremely potent 5-HT_2A_ agonist which induces WDS in rats, although not in a dose-dependent manner. Our data are consistent with other studies concerning NBOMes where an inverted U-shaped dose–response curve for this effect has been observed (Custodio et al. 2019; Elmore et al. 2018). It is possible that when plasma concentration of NBOMes increases, distinct 5-HT receptor subtypes become activated; it was evidenced by Fantegrossi et al. (2010), Vickers et al. (2001), and Klein et al. (2018) that 5-HT_2C_ and 5-HT_1A_ receptors activated by higher concentration of NBOMes modulated their effect on WDS. The data from our study suggest that 25B-NBOMe shares hallucinogenic activity with other classical hallucinogens, such as LSD, DOI, or mescaline. However, it has to be taken into account that many 5-HT_2A_ agonists, such as lisuride, fenfluramine, p-chloromethamphetamine, and L-5-hydroxytryptophan, produce WDS in rats or head twitch response (HTR) in mice, but their effect on this behavior is classified as “false-positive response” (Halberstadt and Geyer 2018). The possible mechanism suggested to differentiate hallucinogenic properties from false-positive effect involves recruitment of different transduction signaling pathways by these groups of compounds (González-Maeso et al. 2007). However, the literature data are not fully conclusive in this respect. The specific effector mechanism responsible for WDS/HTR seems to rely on the G_q/11_-PLCβ cascade resulting in phosphoinositide (PI) hydrolysis and mobilization of intracellular Ca^2+^ ions (Halberstadt and Geyer 2018). However, this signaling cascade is activated by lisuride and other non-hallucinogenic 5-HT_2A_ agonists stronger than by LSD. Furthermore, the study of Moreno et al. (2011) indicates that glutamate mGlu2 receptors and formation of mGlu2 and 5-HT_2A_ receptor heterodimers are required for head twitch behavior. Therefore, other studies are necessary to determine whether WDS/HTR is a valid model of hallucinogenic activity. Regarding hallucinogenic activity of NBOMes, clinical observations and several case studies reported hallucinations after recreational use of 25I-NBOMe (Kyriakou et al. 2015).

### The Effect on Cortical Glutamate Level

Administration of 25B-NBOMe increased extracellular glutamate levels in all studied brain regions as already shown for LSD and DOI (Muschamp et al. 2004; Scruggs et al. 2003). It is consistent with the hypothesis that hallucinogens stimulate cortical glutamate release via 5-HT_2A_ receptor, as shown earlier for DOI, and this effect was blocked by the selective antagonist of this receptor, MDL 100,907 (Scruggs et al. 2003). In our study, the dose of 0.3 mg/kg was the most effective in increasing glutamate release in the frontal cortex, while all the other doses were less potent, but their influence was still significant in comparison to control. Thus, 25B-NBOMe effect on WDS and glutamate release exhibits an inverted U-shaped dose–response curve, just as evidenced earlier for 25I-NBOMe (Herian et al. 2019). The weaker effect of 25I-NBOMe on WDS and glutamate release may be explained by its lower activation potency as shown by Rickli et al. (2015). The inverted U-shaped dose–response curve for inducing glutamate release may be related with negative contribution of 5-HT_2C_ receptors. It is interesting to note that cortical GABA levels were increased by systemic DOI administration (Abi-Saab et al. 1999), which is consistent with the hypothesis that a subset of 5-HT_2C_ receptors located on GABAergic interneurons may modulate the effect of 25B-NBOMe on glutamate release as demonstrated in our study.

### The Effect on Glutamate Level in the Nucleus Accumbens and Striatum

Glutamate release was increased by 0.3 and 3 mg/kg 25B-NBOMe doses in the striatum and nucleus accumbens. The effect was not dose-dependent as the higher dose was weaker in increasing glutamate release in both brain regions. 5-HT_2A_ receptors are broadly expressed not only in the cerebral cortex but also in the nucleus accumbens and caudate nucleus. Receptor binding studies demonstrate relatively high levels of 5-HT_2A_ receptors in the striatum, but its mRNA levels are very low (Bubser et al. 2001). Most 5-HT_2A_ receptors seem to be localized on striatal afferents arising mainly from the cortex and globus pallidus but not in the substantia nigra. It has been suggested that 5-HT_2A_ receptors localized on cortico-striatal axons can regulate glutamatergic activity in the striatum (Ansah et al. 2011). Similarly, 5-HT_2A_ receptors localized on pyramidal cells projecting to the nucleus accumbens may be responsible for glutamate release in this region (Aghajanian and Marek 1997). Modulatory role of 5-HT_2C_ receptors may contribute to a weaker effect of the higher 25B-NBOMe dose on glutamate level in the nucleus accumbens.

### The Effect on DA and 5-HT in the Frontal Cortex

The prefrontal cortex is reciprocally connected with the VTA by dopaminergic afferents and glutamatergic efferents. The observed increase in cortical extracellular DA levels may be mediated through long-distance axons of pyramidal neurons (Di Matteo et al. 2008; Pehek et al. 2006; Soiza-Reilly and Commons 2011) projecting to VTA cells. These axons, by forming synaptic contact with dopamine neurons, may increase DA release by cortical 5-HT_2A_ receptors. This hypothesis was confirmed by studies which showed that 5-HT_2A_ receptor-mediated stimulation of cortico-tegmental projections resulted in enhanced glutamate release in the VTA, which subsequently induced DA efflux in the prefrontal cortex (Kalivas et al. 1989; Kalivas 1993; Pehek et al. 2006). Pyramidal neurons may also participate in control of serotonergic activity. The activation of 5-HT_2A_ receptors localized in layer V pyramidal neurons projecting to the dorsal raphe may be responsible for the hallucinogen-induced increase in cortical 5-HT release (Martín-Ruiz et al. 2001). On the other hand, 5-HT_2C_ receptors expressed on GABAergic cells in the deep layers of the prefrontal cortex may exert inhibitory tone on pyramidal neurons by GABA release. This mechanism may account for a non-linear dose–response curve for DA and 5-HT release observed in our study (Nocjar et al. 2015).

### The Effect on DA and 5-HT in the Nucleus Accumbens and Striatum

Glutamatergic pathways from the prefrontal cortex modulate DA release in the nucleus accumbens by acting on dopamine cells in the VTA (Taber et al. 1995). However, this glutamate pathway does not seem to synapse directly on mesolimbic DA cells. Therefore, an indirect pathway was proposed involving cholinergic neurons located in the pedunculopontine tegmentum (PPT) and/or laterodorsal tegmentum (LDT), which would stimulate dopamine neurons in the VTA projecting to the nucleus accumbens (Semba and Fibiger 1992). Thus, activation through 5-HT_2A_ receptors of both, direct and indirect glutamatergic pathways from the frontal cortex to the nucleus accumbens may be responsible for 25B-NBOMe-induced DA release in this region. A similar effect on DA neurons in the nucleus accumbens for another compound belonging to the same group of psychedelics, i.e., 25I-NBOMe was shown by Miliano et al. (2019). Few studies have evidenced a role of 5-HT_2A_ receptors in nigrostriatal DA release. The 5-HT_2A_ agonist DOI potentiated amphetamine-stimulated DA release in the striatum (Ichikawa and Meltzer 1995; Yamamoto et al. 1995). Furthermore, psilocybin, by binding to 5-HT_2A_ receptors increased striatal DA release in humans (Vollenweider et al. 1999). Thus, this data are in line with our observations that the increase in striatal DA release induced by 25B-NBOMe may be mediated by similar mechanism. The 5-HT_2C_ receptors localized on GABAergic interneurons in the VTA and substantia nigra (Alex and Pehek 2007) and activated by higher doses of 25B-NBOMe may tonically inhibit DA release in the nucleus accumbens and striatum. The cells in the raphe nuclei region are a target of descending glutamatergic pathways from the cortex (Martín-Ruiz et al. 2001). Thus, 25B-NBOMe acting at 5-HT_2A_ receptors located on pyramidal cells increased 5-HT release from neuronal terminals in the nucleus accumbens and striatum. The inverted U-shaped dose–response curve observed in 25B-NBOMe effect on 5-HT release in both brain regions may be explained by inhibitory influence of GABA interneurons activated by higher 25B-NBOMe doses.

The DA striatal/accumbal transmission may also be regulated by ACh, the levels of which are increased in both brain regions. It was reported that cholinergic M_5_ receptors on DA neuronal terminals enhanced DA release, while M_2_/M_4_ autoreceptors on cholinergic terminals inhibited ACh release and subsequent nicotinic nACh receptor-dependent DA release (Shin et al. 2015).

### The Effect on ACh Level in the Frontal Cortex

Basal forebrain projections comprise a majority of cholinergic innervation to the cortex (Lebois et al. 2018). It generally appears that 5-HT exerts a stimulatory influence on the release of ACh (Saito et al. 1996). The effect of stimulation of 5-HT receptor subtypes in vivo remains unclear. In our study, the dose of 0.3 mg/kg of 25B-NBOMe decreased basal extracellular ACh level, but the doses of 0.1 and 10 mg/kg increased cortical ACh levels, while doses of 1 and 3 mg/kg of 25B-NBOMe were not effective. Authors of another study showed that the 5-HT_2A/2C_ agonist DOI as well as mescaline enhanced ACh release in the rat mPFC (Nair and Gudelsky 2004). On the other hand, 5-HT has been shown to inhibit cholinergic neurons in the pedunculopontine and dorsolateral tegmental neurons which express 5-HT_2A_ receptors (Koyama and Kayama 1993). Thus, the effect of 5-HT on the cholinergic system depends upon the receptor localization, making the regulation very complex.

### The Effect on ACh Level in the Nucleus Accumbens and Striatum

The striatum and nucleus accumbens contain numerous cholinergic interneurons (Meredith and Wouterlood 1990). The dose-dependent increase in ACh release in the striatum by 25B-NBOMe, observed in our study, is likely mediated by 5-HT_2A_ receptors (Blomely and Bracci 2005; Bonsi et al. 2007). Moreover, there are DA and ACh relationships in the subcortical brain regions. DA inhibits ACh release acting at D_2_ receptors expressed in cholinergic interneurons (Straub et al. 2014). The lower inhibitory effect of the higher 25B-NBOMe dose on DA striatal levels could be also responsible for a lesser inhibition of ACh release via D_2_ receptors in the rat striatum. On the other hand, inhibition of ACh release by the higher 25B-NBOMe dose in the nucleus accumbens could result from weaker stimulation of glutamate inputs to the nucleus accumbens via cortical D_2_ receptors.

### The Effect on Locomotor Activity in the OF Test

Apart from hallucinogenic activity, psychedelics affect other behaviors. 25B-NBOMe reduced locomotor activity of rats in a dose-dependent manner. The most apparent was a decrease in the number of episodes of walking, crossing, and peeping. This effect was not correlated with changes in concentration of DA, 5-HT, or glutamate as their levels were increased in all studied brain regions. On the other hand, we observed a dose-dependent increase in extracellular ACh levels in the striatum. The GABAergic medium spiny neurons are critical elements in striatal control of animal movement. DA acting through D_2_ receptors modulates striatal cholinergic interneurons (Straub et al. 2014) leading to regulation of the direct and indirect GABAergic pathways (Gerfen and Surmeier 2011) the activity of which is controlled by muscarinic receptors (Threlfell and Cragg 2011). The results of our study may explain neurochemical mechanism underlying DA/ACh interaction in disruption of motor function by 25B-NBOMe.

### The Effect on Anxiolytic/Anxiogenic-like Activity in the LDB Test

Findings of other authors also showed that phenylethylamine and indoleamine hallucinogens such as mescaline, DOI, DOM or LSD, DMT, and psilocin reduced locomotor activity of rats in unfamiliar environment (Halberstadt and Geyer 2018). It has been suggested that this effect reflects the fear in novel settings and increased center avoidance induced by hallucinogens reminding agoraphobia observed in humans. Our results from the light/dark box test are in agreement with the above findings. In our study, 25B-NBOMe prolonged the time spent by animals in the dark zone, while it decreased the time spent in the light zone. These data suggest that acute 25B-NBOMe doses are likely to induce anxiety in animals. In addition, the decreased ambulatory distance, vertical and stereotypical activity time demonstrates that motor activity of rats was suppressed. This data confirm our findings in the open field test showing a decreasing effect of 25B-NBOMe on exploration of animals.

Several neurotransmitters may be involved in the anxiogenic effect of 25B-NBOMe, which are released into the synaptic cleft after stimulation of serotonin 5-HT_2A_ receptors. Among the most important neurotransmitters which play the role in anxiety, there are GABA, glutamate, 5-HT, and ACh. GABA system regulates neuronal excitability and attenuation of GABAergic system results in anxiety (Nemeroff 2003). Glutamate, as a main excitatory neurotransmitter in the CNS has been shown to play an important role in different brain functions, *inter alia* stress and anxiety (Meldrum 2000). In particular, glutamate in limbic system plays a pivotal role in the pathogenesis of anxiety disorders (Bergink et al. 2004). 5-HT is another important player in the development of anxiety disorders, and an increase in 5-HT concentration in the brain also increases anxiety (Graeff 2002). A role of 5-HT in anxiety is supported by its modulating effect on the locus coeruleus (LC), while fear and stress activate serotonergic pathways (Akimova et al. 2009; Graeff 2002). Moreover, ACh which is mainly engaged in memory and learning processes can be modulated by stress (Deepak et al. 2012), and activation of cholinergic M_1_ receptors induce anxiety through noradrenergic pathways (Mineur et al. 2013). In our study, we observed changes in the abovementioned neurotransmitters, important in generating the anxiety. 25B-NBOMe being a very potent 5-HT_2A_ receptor agonist, by excitation of cortical pyramidal cells increases glutamate release not only in the frontal cortex, but indirectly, through descending neuronal pathways, in the striatum and nucleus accumbens. Thus, the rise in glutamate release seems to be the main cause of anxiogenic effect of 25B-NBOMe in the LDB test. Furthermore, the increase in 5-HT and ACh release from neuronal terminals in the studied brain regions may be strongly responsible for anxiogenic behavior observed in LDB test. The modulatory impact of GABA resulting from activation by 25B-NBOMe of 5-HT_2C_ receptors located on GABAergic interneurons seems to be less effective in this test; therefore, linear dose–response is observed in this effect.

### The Effect on Cognitive Functions in the NOR Test

ACh is the major neurotransmitter involved in memory, and cholinergic systems in the cortex, striatum, and nucleus accumbens are implicated in cognitive functions (Lebois et al. 2018; Woolf and Butcher 2011). It is known that reduction in the central cholinergic system function occurs in dementia and Alzheimer’s disease (Bartus et al. 1982). In our study, we observed different changes in cortical ACh release depending on the 25B-NBOMe dose. ACh release was decreased by the dose of 0.3 mg/kg, and no effect was seen after the dose of 3 mg/kg. The extracellular ACh levels were increased in the striatum and nucleus accumbens, but the response to 25B-NBOMe was dose-dependent only in the striatum. The higher dose of 25B-NBOMe was weaker in increasing ACh level than the lower one in the nucleus accumbens. Interestingly, the exploration of novel object in the NOR test was disturbed by the higher dose of 25B-NBOMe. This observation suggests that 25B-NBOMe may disturb memory processing linking this effect with the modulation of ACh levels in the nucleus accumbens.

In order to study further the role of ACh neurons in the NOR test, rats were co-injected with the non-selective muscarinic receptor antagonist scopolamine. Scopolamine increased exploration time and recognition index decreased by 25B-NBOMe, had no effect on motor activity in the OF test, reduced by 25B-NBOMe, but increased the time of walking alone when compared to control. These data strongly indicate that neuromodulatory ACh system is involved in memory and motor functions and that ACh modulates effects of serotonin receptors activated by 25B-NBOMe. The primary cholinergic input to the cerebral cortex which comes from the basal forebrain complex (Mesulam 1995) activates the pyramidal cells by M_1_ ACh receptors or via M_2_ receptors located on GABAergic interneurons (Picciotto et al. 2012). ACh also suppresses cortico-cortical transmission through M_2_ receptors expressed in pyramidal cell axon terminals (Picciotto et al. 2012). The striatum and its ventral part, nucleus accumbens, contain cholinergic interneurons. The complex synaptic effects of ACh provide mechanism for the ability of ACh to modulate cognitive behaviors. Depending on which receptor is recruited, cortical ACh transmission may generate different responses. Scopolamine commonly used to induce cognitive deficit in animals, in our hands improved rats’ behavior in the NOR test. This unexpected effect may be related with neuromodulatory role played by ACh in the CNS. ACh may diffuse within extracellular space through volume transmission on long distances to reach extra-synaptic receptors and may be also co-released with other neurotransmitters, e.g., glutamate or GABA (Colangelo et al. 2019). The observed changes in DA, glutamate, and 5-HT neurotransmission under influence of 25B-NBOMe can be modulated by ACh, and switching between different behavioral states in the presence of scopolamine may occur. The findings of Day et al. (1991) and Durkin et al. (1992) showed an increase in ACh release in the hippocampus, striatum, and frontal cortex of rats and increase in locomotor activity by scopolamine administration. On the other hand, the impaired novel object discrimination by scopolamine was reported by Ennaceur and Meliani (1992). The motor coordination in mice was decreased, and profound deficits in attention and memory were observed in mice treated with scopolamine (Falsafi et al. 2012). Furthermore, increased levels of M_1_ and NMDA receptors co-localized in hippocampal pyramidal cells were observed in scopolamine treated animals. It is suggested that interaction between M_1_ and NR1 subunit of NMDA receptor is essential for memory formation and is modified by scopolamine (Falsafi et al. 2012). It is proposed that the central cholinergic system modulates the excitatory transmission and that ACh stimulation of muscarinic receptors potentiates responses of NMDA. Our behavioral data suggest that scopolamine could modulate the response of cholinergic neurons to 25B-NBOMe treatment leading to improvement of cognitive deficit induced by this hallucinogen. However, the exact mechanism of this behavioral impairment needs further studies.

The alternative explanation of results in NOR and other behavioral tests is grounded on an alteration of preference of rats for novelty. Psychedelic drugs have profound effects on the response to novel stimuli. When rats are tested in novel environment, psychedelic drugs alter exploratory and investigatory behavior but have no effect in a familiar environment. The locomotor activity and investigatory behavior observed in the OF test and NOR test have been markedly attenuated by 25B-NBOMe and likely reflected potentiation of the neophobia exhibited by rats in novel settings (Adams and Geyer 1985; Mittman and Geyer 1991; Wing et al. 1990). Reduction of locomotor activity by hallucinogens in a novel environment, but no effect or increase in a familiar environment, has been reported by other researchers (Hillegaart et al. 1996; Ouagazzal et al. 2001; Tilson et al. 1975). In humans, LSD or other hallucinogens markedly enhance reactivity to unpleasant or threatening stimuli (Cohen 1960). Thus, the increased avoidance of novel and open areas observed in rats after administration of hallucinogens may be analogous to the enhanced reactivity to environmental stimuli observed in humans. Notably, psychedelic drugs markedly enhance the ability of peripheral stimuli to activate the LC (Aghajanian 1980; Rasmussen and Aghajanian 1986), a brain region that functions as a novelty detector. Regional distribution of 5-HT_2A_ and all subtypes of α1-adrenergic receptors are very similar in deep layers of the prefrontal cortex and mRNA of both receptors is abundant in pyramidal and GABAergic neurons (Nichols 2016). Systemic but not local administration of hallucinogens to anaesthetized rats decreased spontaneous activity of LC cells but enhanced the activity of LC neurons evoked by sensory stimuli (Aghajanian 1980; Rasmussen and Aghajanian 1986). As LC sends noradrenergic projections to the cortex, changes in LC firing would also affect pyramidal cells activity. LC reactivity may underlie the mechanism of hallucinogen-induced neophobia, reduction of locomotor activity and fear exhibited by rats in novel settings as observed in the OF and NOR test.

### Neurotoxicity

The observed increase in extracellular DA and glutamate levels may imply neurotoxic effect of NBOMe compounds. In fact, in vitro cytotoxic activity of 25B-NBOMe was demonstrated in primary rat cortical cultures (Zwartsen et al. 2019). However, 25B-NBOMe tested in the comet assay showed only minor damaging effect on DNA in the nuclear fraction from the rat frontal cortex. Interestingly, the 25B-NBOMe doses of 0.3 and 3 mg/kg produced some damage of nuclear DNA which was inversely correlated with the dose. In contrast, MDMA at a dose of 10 mg/kg used as the reference drug induced potent oxidative DNA damage. Oxidative stress and excitotoxicity represent mechanisms causing neuronal damage by MDMA (Cadet et al. 2001). MDMA at the dose of 10 mg/kg has been shown to augment DA and 5-HT release in several regions of rat brain, which may be a source of oxidative stress (Gołembiowska et al. 2016). DNA single- and double-strand breaks were observed in rat and mouse cortex after administration of MDMA (Frenzilli et al. 2007; Górska et al. 2018; Noworyta-Sokołowska et al. 2016). In the present study, the tissue contents of DA, its metabolites DOPAC and HVA and 5-HT and 5-HIAA were not affected by 25B-NBOMe (Table 1 in supplementary data). Similarly, any damaging effect was observed in our recent study with 25I-NBOMe (Herian et al. 2019). It is unclear why the drugs displaying so profound effects on several brain neurotransmitters, particularly on DA, 5-HT, and glutamate extracellular levels are slightly neurotoxic when tested in vivo. However, it is obvious that increased release of glutamate by 5-HT_2A_ agonists from pyramidal cells is accompanied with GABA release from GABAergic interneurons. This effect balances excitation of pyramidal neurons driven by released glutamate (Beique et al. 2007). Our neurochemical data showing the dose of 0.3 mg/kg 25B-NBOMe as the most potent in enhancing glutamate, DA and 5-HT release stays in accordance with the pronounced damage of nuclear DNA caused by this dose. The lack or very weak neurotoxic effect of this class of hallucinogens is in contrast to indoleamine hallucinogens. In our earlier work, we have shown that tryptamine derivative, 5-MeO-DIPT, induced genotoxicity in the comet assay and affected caspase-3 activity and enzymatic defense system (Noworyta-Sokołowska et al. 2019). It is suggested that the underlying mechanism of tryptamine hallucinogen neurotoxicity involves oxidative stress generated by profound accumulation of DA and tryptamine oxidative products and excitotoxicity (Górska et al. 2018). Therefore, other methods need to be used to understand the gap between the in vitro cytotoxic action of NBOMe compounds and the lack or very weak damaging effect in vivo.

## Conclusions

In summary, administration of 25B-NBOMe, a potent agonist of 5-HT_2A/C_ receptors, facilitated DA, 5-HT, glutamate, and ACh release in the rat frontal cortex, striatum, and nucleus accumbens. The enhancement of neurotransmitter levels seems to be regulated in an opposite manner by 5-HT_2A_ and 5-HT_2C_ receptors, which was reflected by a U-shaped dose–response curve. The increased cortical glutamate release may be responsible for hallucinogenic activity of 25B-NBOMe and increased cortico-striatal and cortico-accumbal neurotransmission. The impaired attention and motor activity may depend on changes in extracellular ACh levels in the nucleus accumbens and striatum, respectively. Prolongation of the time spent in the dark zone suggests anxiogenic effect of this compound. In spite of in vitro cytotoxic activity, the in vivo data do not indicate that 25B-NBOMe is evidently neurotoxic.

## Electronic Supplementary Material

Below is the link to the electronic supplementary material.

Supplementary file1 (DOC 4.04 MB)

Supplementary file2 (DOC 106 kb)

Supplementary file3 (DOC 36.5 kb)
