# Supplementary material for: Neurochemical and Behavioral Effects of a New Hallucinogenic Compound 25B-NBOMe in Rats
Source: Neurotox Res. 2020 Dec 18;39(2):305–26. doi: 10.1007/s12640-020-00297-8 (PMC7936972; doi:10.1007/s12640-020-00297-8)
Supplement: Supplementary file 2 — Supplementary file2 (DOC 106 kb) [file 12640_2020_297_MOESM2_ESM.doc]

**Fig. 2s.** The effect of scopolamine on 25B-NBOMe (0.3 and 3 mg/kg)-induced changes in rats’ performance in the novel object recognition (NOR) test. The exploration time of two identical objects is shown in the introductory session. Values are the mean ± standard error of the mean (SEM), n = 6-12 per experimental group. # *p* < 0.05, ## *p* < 0.01 vs. control; * p < 0.01 A1 vs. A2 objects; $ *p* < 0.01 vs. scopolamine (one-way ANOVA and Tukey’s post hoc test).
