# Supplementary material for: Neurochemical and Behavioral Effects of a New Hallucinogenic Compound 25B-NBOMe in Rats
Source: Neurotox Res. 2020 Dec 18;39(2):305–26. doi: 10.1007/s12640-020-00297-8 (PMC7936972; doi:10.1007/s12640-020-00297-8)
Supplement: Supplementary file 3 — Supplementary file3 (DOC 36.5 kb) [file 12640_2020_297_MOESM3_ESM.doc]

**The measurement of tissue contents of DA, 5-HT and their metabolites**

Animals were sacrificed by decapitation 3 h after subcutaneous drug administration. Brains were removed and frontal cortices were dissected in anatomical borders. The tissue levels of DA, 5-HT, 3,4-dihydroxyphenylacetic acid (DOPAC), homovanillic acid (HVA), and 5-hydroxyindoleacetic acid (5-HIAA) were measured using a high-performance liquid chromatography (HPLC) with electrochemical detection. Tissue samples of frontal cortices were homogenized in an ice-cold 0.1 M HClO_4_, and were centrifuged at 10,000 x g for 10 min at 4 °C. The obtained supernatants were filtered through 0.22μm Ultrafree Centrifugal Filters (Merck Millipore Ltd., Irleand) and 3-5 μL of samples were injected into an HPLC system. The chromatographic system consisted of an UltiMate 3000 pump (Thermo Scientific, USA), an LC-4C amperometric detector with a cross-flow detector cell (BAS, IN, USA), and a HR-80 analytical column (3 μm, 80 × 4.6 mm, ESA, Inc., USA). The mobile phase was composed of 0.1 M KH_2_PO_4_, 0.5 mM EDTA, 95 mg/L sodium 1-octanesulfonate, and 4% methanol, adjusted to pH=3.8 with 85% H_3_PO_4_. The flow rate was 1 mL/min. The potential of a 3-mm glassy carbon electrode was set at +700 mV with sensitivity of 5 nA/V. The temperature of the column was maintained at 30°C. The obtained data were collected and processed by Chromax 2007 software (Pol-Lab, Warszawa, Poland).

**Table 1** Tissue contents of DA, DOPAC, HVA, 5-HT, and 5-HIAA in the frontal cortex measured 3 h after administration of 25B-NBOMe

| Treatment (mg/kg) | DA | DOPAC | HVA | 5-HT | 5-HIAA |
| --- | --- | --- | --- | --- | --- |
| Frontal cortex pg/mg wt ± SEM (n) | | | | | |
| Control | 482 ± 43 (8) | 129 ± 15 (8) | 110 ± 10 (8) | 514 ± 39 (8) | 257 ± 11 (8) |
| 25B-NBOMe 0.3 | 428 ± 38 (8) | 114 ± 21 (8) | 104 ± 18 (8) | 444 ± 45 (8) | 205 ± 17 (8) |
| 25B-NBOMe 1 | 465 ± 37 (8) | 108 ± 7 (8) | 109 ± 16 (8) | 483 ± 50 (8) | 230 ± 20 (8) |
| 25B-NBOMe 3 | 419 ± 50 (8) | 113 ± 21 (8) | 99 ± 8 (8) | 454 ± 49 (8) | 201 ± 18 (8) |
| 25B-NBOMe 10 | 496 ± 39 (8) | 106 ± 7 (8) | 103 ± 4 (8) | 493 ± 53 (8) | 226 ± 21 (8) |

*P* > 0.05 (one-way ANOVA and Tukey’s post hoc test)
